# Characterization of the Mechanical and Morphological Properties of Cow Dung Fiber-Reinforced Polymer Composites: A Comparative Study with Corn Stalk Fiber Composites and Sisal Fiber Composites

**DOI:** 10.3390/polym14225041

**Published:** 2022-11-21

**Authors:** Siyang Wu, Mingzhuo Guo, Jiale Zhao, Qian Wu, Jian Zhuang, Xinming Jiang

**Affiliations:** 1 College of Biological and Agricultural Engineering, Jilin University, 5988 Renmin Street, Changchun 130022, China; 2 Key Laboratory of Bionic Engineering, Ministry of Education, Jilin University, 5988 Renmin Street, Changchun 130022, China; 3 College of Engineering and Technology, Jilin Agricultural University, 2888 Xincheng Street, Changchun 130118, China

**Keywords:** polymer matrix composites, cow dung waste, natural fiber, mechanical properties, interface performance

## Abstract

Natural fibers and their composites have attracted much attention due to the growing energy crisis and environmental awareness. In this work, a natural lignocellulosic fiber was extracted from cow dung waste and its potential use as reinforcing material in resin-based polymer composites was evaluated. For this purpose, cow dung fiber-reinforced composites (CDFC) were fabricated, and their mechanical and morphological properties were systematically investigated and compared with corn stalk fiber composites (CSFC) and sisal fiber composites (SFC). The results showed that the addition of cow dung fibers reduced the density of the polymer composites, increased the water absorption, and enhanced the impact strength and shear strength. The highest impact and shear strengths were obtained at 6 wt.% and 9 wt.% of fiber loading, respectively, which increased by 23.8% and 34.6% compared to the composite without the fibers. Further comparisons revealed that at the same fiber addition level, the CDFC exhibited better mechanical properties than the CSFC; notably, the CDFC-3 (adding 3 wt.% of fiber loading) had an impact strength closer to the SFC-3. Furthermore, an SEM analysis suggested that the cow dung fibers exhibited a rough and crinkly surface with more node structures, and presented good interfacial bonding with the composite matrix. This work revealed that cow dung fibers are a promising candidate as reinforcement for resin-based polymer composites, which promotes an alternative application for cow dung waste resources in the automotive components field.

## 1. Introduction

The increasing energy crisis and environmental issues in recent years have prompted global researchers to develop green and sustainable fiber-reinforced polymer composites for the automotive, aircraft, and marine industries [1,2,3]. In general, the fibers used in polymer composites for engineering applications mainly include synthetic fibers and natural fibers. Synthetic fibers, such as carbon fiber and glass fiber, have the advantages of being lightweight and high strength, and have excellent mechanical properties, corrosion resistance, fatigue resistance, and so on, making synthetic fiber composites the main load-bearing components in engineering fields. However, the preparation these synthetic fibers has an adverse impact on the environment, and they are nonrenewable materials with high costs. In recent years, natural fibers have gradually emerged as an attractive alternative to synthetic fibers with respect to their ecological and economic advantages [4,5,6,7]. In fact, natural fibers, as typical renewable and eco-friendly materials, not only endow polymer composites with biodegradability and nontoxicity, but also reduce their weight, have significant cost benefits, and produce low carbon emissions [8,9,10,11,12]. The natural fibers used in polymer composites are mainly derived from various plant fibers, including leaf, bast, stalk, seed, grass, wood, etc. [13]. Recently, many efforts have been made by researchers to develop and evaluate polymer composites reinforced with different plant fibers, such as sisal, jute, bamboo, flax, corn stalk, coir, rattan, hemp, and betelnut fibers [14,15,16,17,18,19]. Intrinsically, these plant fibers, as lignocellulosic biomass, are mainly composed of cellulose, hemicellulose, and lignin, which give them specific properties, such as relatively high specific strength and stiffness [20,21].

Cow dung is a readily available biomass resource and is considered to contain a high proportion of lignocellulosic material due to the special digestive action of cows [22,23]. As a kind of ruminant, a cow has a complex stomach consisting of four chambers: the reticulum, rumen, omasum, and abomasum [24]. During digestion, the raw materials are first roughly chewed and swallowed into the rumen chamber and reticulum chamber, and the partly digested materials (cud) are then returned from the two chambers to the mouth. After being rechewed and reswallowed, the materials finally enter the omasum chamber and abomasum chamber, which squeeze out most of the moisture [25]. These stomach chambers contain a series of microorganisms, such as bacteria, fungi, protozoa, and trichomonas, which are capable of digesting plant fibers, especially hemicellulose and pectin components, through enzymatic hydrolysis [22]. As a consequence, a large percentage of cellulose is undigested and preserved in the cow dung [26,27]. 

Cows are the second most populous livestock in the world, with a population of about 1000 million in 2021 [22]. A mature cow generally produces about 15 kg of dung a day, and it is estimated that the daily production of cow dung is up to approximately 15 million tons [28]. However, the utilization of cow dung is insufficient, especially in China, where more than 50% of cow dung is directly piled up around farms. Moreover, due to the slow decomposition of cow dung and the release of harmful gases from the original feces, this not only leads to the waste of renewable biomass resources but also causes pollution of soil, water, and air [29]. In this respect, it is of great significance to effectively utilize cow dung waste resources and convert them into value-added products. 

In recent years, studies have focused on the recycling and utilization of cow dung waste, including to sequester heavy metals from wastewater [30], to stabilize subgrade soil [31], as a source for biogas production [32], composting [33], and low-cost thermal insulation [34], as fermentation media for enzyme production [35], as a reductant for the reduction roasting of iron ore slime [28], as a component in microbial safety control [36], and so on. In addition, various researchers have used cow dung as a biofiller in composite materials. Li et al. [22] introduced cow dung waste into cementitious composites and characterized their mechanical and autogenous shrinkage properties. This work revealed that the pretreated cow dung fibers increased the splitting tensile strength and compressive strength and reduced the autogenous shrinkage of the composites. Vedrtnam [37] fabricated a novel composite film using cow dung and polyvinyl alcohol for packaging applications. It was found that adding 6% cow dung fibers improved the composite film’s flexural and tensile strengths but reduced the thermal conductivity. However, so far, due to the limitations of technology and capital, cow dung waste resources have not been applied on a large scale. Therefore, more research is imperative to explore the potential applications of cow dung waste. 

Recently, the related literature has shown that natural lignocellulosic fibers can be used as reinforcement materials in polymer composites [38,39,40,41,42]. Cow dung fibers belong to the group of natural lignocellulosic fibers. Since most of the cows in China feed on corn stalks, the cow dung fibers are somewhat similar to biologically treated corn stalk fibers. Thus, in this study, an attempt is made to explore the reinforcement potential of cow dung fibers in polymer matrix composites. For this purpose, the cow dung fibers were first separated from cow dung waste, and characterized for their morphological, physical, and chemical properties. Then, the cow dung fiber-reinforced composites (CDFC) were fabricated, and their mechanical behaviors, including density, water absorption, impact strength, and shear strength, were investigated. In order to better evaluate the reinforcing effect of cow dung fibers, the polymer composites reinforced with corn stalk fibers (CSFC) and sisal fibers (SFC) were also prepared using the same process, and the performances of these three polymer composites (CDFC, CSFC, and SFC) are compared and discussed. In addition, the fracture surfaces of the polymer composites after mechanical testing were determined and analyzed using a scanning electron microscope. The obtained results not only promote an alternative application for cow dung waste resources but also provide a sustainable reinforcement material for biopolymer composites.

## 2. Materials and Methods

### 2.1. Fiber Preparation

**Figure 1:**
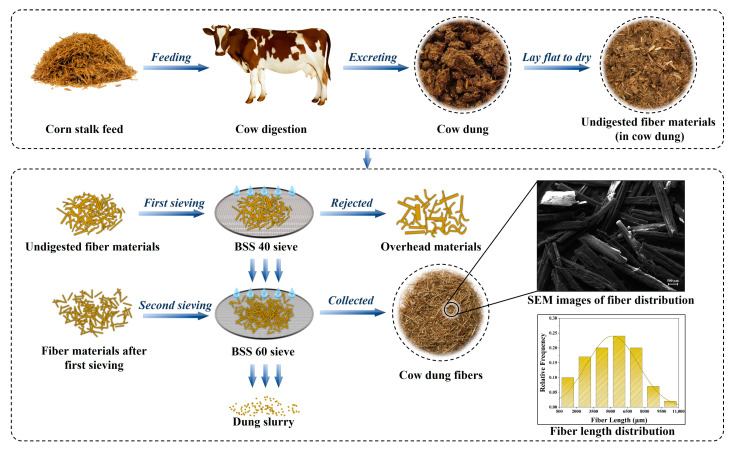
The schematic diagram of the preparation process of the cow dung fibers.

The cow dung used in the present work was collected from a local farm in Changchun, China. The cows were fed on corn stalks as their staple food, resulting in a lot of stalk fibers in the feces. In order to separate the solid fibers from the feces, the collected cow dung was passed through 40-mesh and 60-mesh sieves in sequence under normal tap water flow. The materials that did not pass through the 40-mesh sieve were rejected due to the presence of large impurities, and the materials that passed through the 40-mesh sieve and were retained on the 60-mesh sieve were selected as cow dung fibers for further treatment. The schematic diagram of the preparation process of the cow dung fibers (CDF) and the corresponding fiber length distribution are shown in Figure 1. These fiber samples were treated with 2 wt.% NaOH solution for 40 min and then washed repeatedly with distilled water until reaching a neutral pH value. After that, the fiber samples were dried at 70 °C for at least 24 h using a vacuum drying oven (ZK350S, Tianjin, China). The corn stalks used as cow feed were collected from a local farm in Changchun, China, and the sisal fibers used for the comparison test were purchased from Zhanjiang Dongfang Sisal Group Co., Ltd., Zhanjiang, China. The corn stalk fibers (CSF) and sisal fibers (SF) were processed to a fiber size similar to that of the CDF and were subjected to alkali treatment using the same process as described above.

### 2.2. Fiber Morphology Characterization

A scanning electron microscope (SEM, EVO-18, Zeiss, Germany) was used to observe the surface morphology of the CDF, CSF, and SF and to evaluate their morphological differences. The SEM analysis was carried out at a 15 kV accelerating voltage. Prior to characterization, the fiber samples were gold-coated in a sputtering coater (SBC-12, Zhongke Instruments, China) to enhance their surface conductivity.

### 2.3. X-ray Diffraction (XRD) Measurement

An X-ray diffractometer (D/max 2500pc, Rigaku, Japan) was used to determine the cellulose structure and the degree of crystallinity of the CDF, CSF, and SF. The XRD analysis was carried out at a 40 kV voltage and a 30 mA current. The XRD patterns of these fiber samples were recorded in the 2θ angle range from 5° to 60° with a sampling pitch of 0.02°. The crystallinity index (*CrI*) of the fiber samples was determined by Equation (1), according to the Segal empirical method [43].

(1) $$CrI=\frac{I_{200}-I_{\mathrm{am}}}{I_{200}}\times 100\%$$

where *I*_200_ and *I*_am_ are the intensity of the cellulose peak (at a 2θ angle of 22–23°) and the amorphous peak (at a 2θ angle of 18–19°), respectively.

### 2.4. Fourier Transform Infrared Spectroscopy (FTIR) Measurement

A Fourier transform infrared spectrometer (Equinox 55, Bruker, Germany) was used to investigate the functional groups present in the CDF, CSF, and SF and to characterize their component changes. The FTIR spectra of these fiber samples were recorded at the scanning wavenumber range from 400 cm^−1^ to 4000 cm^−1^ with a resolution of 4 cm^−1^.

### 2.5. Preparation of the Fiber-Reinforced Polymer Composites

The composition details of the prepared polymer composites are given in Table 1. The composites containing cow dung fibers, corn stalk fibers, and sisal fibers were designated as CDFC, CSFC, and SFC, respectively, and the composite without the addition of these three fibers was used as the reference, designated as Ref. In this study, the fiber-reinforced polymer composites were manufactured using the hot press molding technique. During the manufacturing, the raw materials were thoroughly mixed in a paddle-type blender (JF805R, Wanda Machinery, Changchun, China) for 10 min at room temperature, and then the mixtures were hot-pressed in a thermocompressor (JFY50, Wanda Machinery, Changchun, China) for 30 min at 165 °C and 40 MPa. After that, the obtained composites were heat-treated in a heat-treatment oven (JF980B, Wanda Machinery, Changchun, China) and were machine-cut to specific sizes for the following tests. The detailed preparation process of the polymer composites was described in our previous work [16,40,44].

### 2.6. Density Test

The density of the Ref, CDFC, CSFC, and SFC was determined by a precision electronic balance (MP5002, Shanghai, China) based on the Archimedes method. The density (*ρ*) values of these polymer composites were calculated according to Equation (2) [45].

(2) $$\rho =\frac{m_{1}}{m_{1}-m_{2}}\times \rho_{w}$$

where *m*_1_ and *m*_2_ are the dry weight of the polymer composites in air and the soaked weight in distilled water, respectively, and *ρ*_w_ is the density value of the distilled water (1 g·cm^−3^).

### 2.7. Water Absorption Test

The water absorption of the Ref, CDFC, CSFC, and SFC was measured according to the standard GB/T 24508-2009 [46]. Prior to testing, the polymer composites were dried in a vacuum drying oven at 70 °C to obtain a constant mass. Then, these composites were submerged in distilled water at room temperature for 72 h. Next, the composites were dried using tissue paper to remove water from the surface. Then, the composites were immediately weighed, and the water absorption (*WA*) values were calculated by Equation (3).

(3) $$WA=\frac{M_{1}-M_{0}}{M_{0}}\times 100\%$$

where *M*_0_ and *M*_1_ are the weight of the polymer composites before and after water immersion, respectively.

### 2.8. Mechanical Test

The mechanical tests for the polymer composites in this study included an impact strength test and shear strength test, according to the standards GB/T 33835-2017 and GB/T 26739-2011, respectively. The impact strength of the Ref, CDFC, CSFC, and SFC was carried out by an impact tester (XJ-40A, Shanghai, China) to assess the impact-resistance properties and evaluate the overall composite toughness. The tested polymer composites were prepared to a standard size of 55 mm × 6 mm × 4 mm. The shear strength was measured by a universal testing machine (WAW-100, Guilin, China) to determine the ability of these composites to resist shear loads, and the tests were performed at a crosshead speed of 10 mm/min with a sample size of 20 mm × 20 mm × 10 mm. The average of five parallel tests for each composite was taken as the final result.

### 2.9. Interfacial Morphology Analysis

The fracture surfaces of the tested polymer composites after the mechanical tests were observed by using SEM under an accelerating voltage of 20 kV. Prior to SEM analysis, these polymer composites were sputter-coated with a thin layer of gold.

### 2.10. Statistical Analysis

The statistical data in this paper were analyzed by the least significant difference (LSD) method using the Origin software. LSD was mainly used to determine whether there were significant differences among the different levels of one test factor and to compare the averages of different levels. The mean and standard deviation of the test indicators (including the density, water absorption, impact strength, and shear strength) were obtained from five parallel tests.

## 3. Results and Discussion

The discussions in this section mainly consist of two parts: the properties of cow dung fibers, including the SEM analysis, XRD analysis, and FTIR analysis, and the properties of cow dung fiber-reinforced resin-based polymer composites, including density, water absorption, impact strength, shear strength, and fracture surface analysis. To better evaluate the reinforcement potential of cow dung fibers, we further compared cow dung fiber composites with corn stalk fiber composites and sisal fiber composites. The detailed results and discussions are as follows.

### 3.1. SEM Analysis

**Figure 2:**
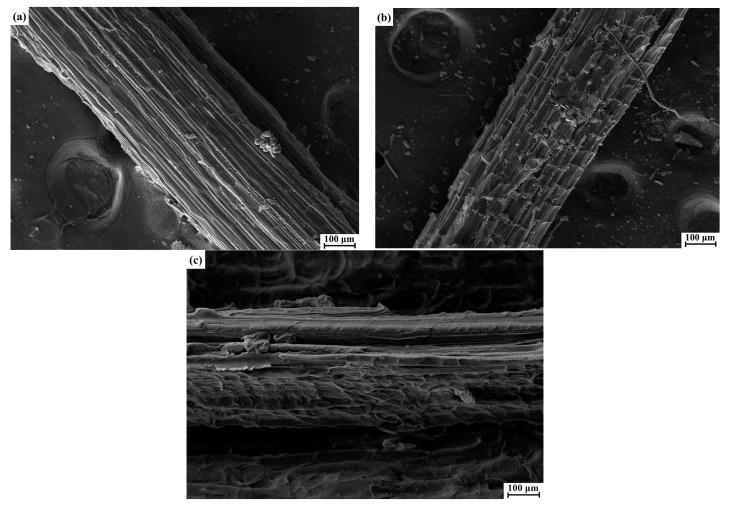
SEM images of the (**a**) CSF, (**b**) CDF, and (**c**) SF.

The SEM micrographs of the CDF, CSF, and SF are shown in Figure 2. It can be clearly seen that there were some morphological differences between these three fibers. The surface of the CSF, as given in Figure 2a, was relatively smooth and even, with a regular structure. The CDF presented a rough and crinkly surface texture (Figure 2b), due to the partial removal of impurities, including hemicellulose and lignin, after the digestion treatment by the cow stomach [29,47]. Moreover, more node structures were also observed on the CDF surface. These features contribute to increasing mechanical interlocking, resulting in a better interfacial interaction between the fibers and the composite matrix [16,48]. As for the SF, as shown in Figure 2c, its surface appeared relatively dense and uneven, which was mainly because the sisal fibers had a higher content of cellulose. Similar observations were also found by Yan et al. [49].

### 3.2. XRD Analysis

**Figure 3:**
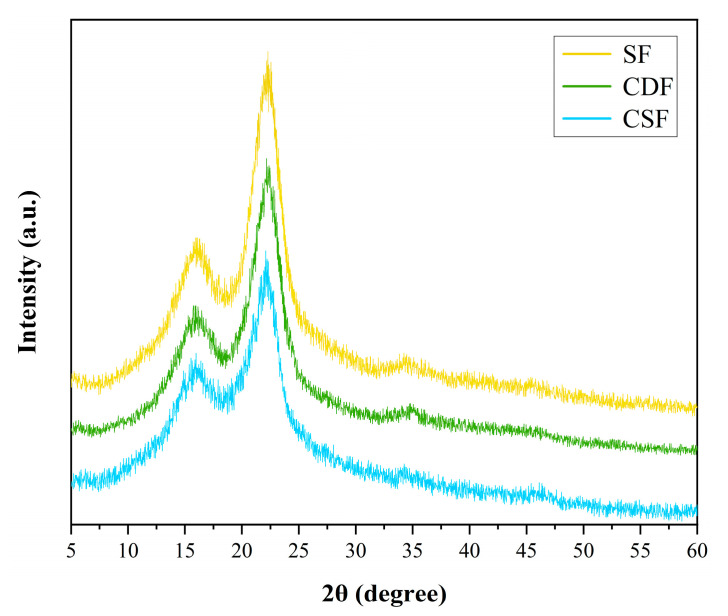
XRD patterns of the fiber samples CDF, CSF, and SF.

The diffraction patterns and *CrI* values of the CDF, CSF, and SF are shown in Figure 3 and Table 2, respectively. It can be observed from Figure 3 that the XRD patterns of CDF, CSF, and SF were basically the same, and they presented the main and secondary diffraction peaks at 2θ angles of around 22° and 16°, corresponding to the cellulose crystallographic plane and amorphous component reflection, respectively. This also indicated that the three natural fibers in this study exhibited almost the same cellulose crystallites (type I), and no structural transformation occurred after the digestive treatment of the cow. Further, comparing the crystallinity degrees of these fiber samples, as shown in Table 2, the *CrI* value of CDF (57.69%) was higher than that of CSF (51.53%), which may be due to the enzymatic hydrolysis of amorphous parts, including hemicellulose and lignin, during the digestion process [24,47]. However, the relative crystallinity of the CDF was lower than that of the SF (61.77%), owing to the higher cellulose content in sisal fibers, as reported in the published literature [13].

### 3.3. FTIR Analysis

**Figure 4:**
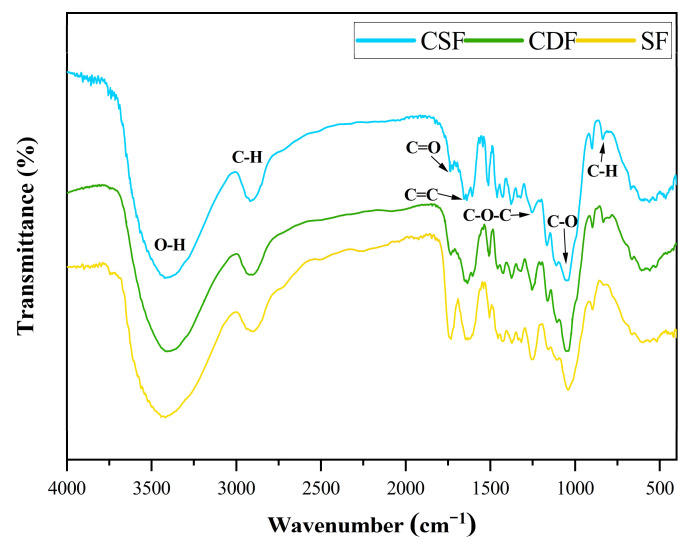
FTIR spectra of the fiber samples CDF, CSF, and SF.

The FTIR spectra of the CDF, CSF, and SF are shown in Figure 4. As observed from Figure 4, the three fibers in this study exhibited similar patterns with notable bands, which were characteristic of lignocellulosic fibers, but the intensity of some absorbance bands differed in these fibers. The broad absorption peak at 3398 cm^−1^ in the CDF, 3385 cm^−1^ in the CSF, and 3417 cm^−1^ in the SF corresponded to the O–H stretching vibration of the cellulose. The peak at 2914 cm^−1^ in the CDF, 2906 cm^−1^ in the CSF, and 2902 cm^−1^ in the SF was related to the C–H stretching vibration of the CH and CH_2_ in the hemicellulose and cellulose. The peak at 1732 cm^−1^ in the CSF was associated with the C=O stretching vibration of the hemicellulose, and its absorbance was lower in the CDF and SF, indicating the lower hemicellulose content in the cow dung fibers and sisal fibers compared to the corn stalk fibers. The peak at 1604 cm^−1^ in the CSF belonged to the C=C stretching vibration of the lignin, and the intensity of this peak decreased in the CDF due to the partial removal of the lignin after cow digestion. It was even more reduced in the SF owing to less lignin content in the sisal fibers. The peaks at 1506 cm^−1^ and 1419 cm^−1^ in the CDF, 1508 cm^−1^ and 1423 cm^−1^ in the CSF, and 1506 cm^−1^ and 1421 cm^−1^ in the SF were attributed to the benzene skeleton vibration of the lignin. The peak at 1242 cm^−1^ in the CDF, 1249 cm^−1^ in the CSF, and 1246 cm^−1^ in the SF was related to the C–O–C stretching vibration of the cellulose, and the peak intensity was higher in the SF, followed by the CDF and CSF, suggesting a higher cellulose content in the sisal fibers. The peak at 1037 cm^−1^ in the CDF, 1039 cm^−1^ in the CSF, and 1049 cm^−1^ in the SF was ascribed to the C–O stretching vibration in the hemicellulose and cellulose. The peak at 896 cm^−1^ in the CDF, 894 cm^−1^ in the CSF, and 896 cm^−1^ in the SF belonged to the β-glycosidic linkages between the monosaccharides. The peak at 831 cm^−1^ in the CSF was due to the C–H stretching vibration out of the aromatic phenyl ring plane in the lignin, which became weaker in the CDF and almost disappeared in the SF; this also indicated that the lignin content of the cow dung fibers and sisal fibers was lower than that of the corn stalk fibers [46,50,51,52].

### 3.4. Density Analysis

**Figure 5:**
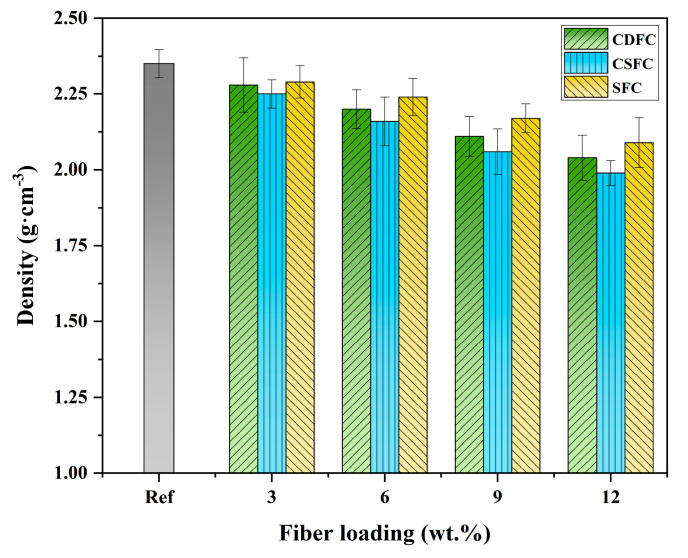
Density of the polymer composites Ref, CDFC, CSFC, and SFC.

The density results of the polymer composites Ref, CDFC, CSFC, and SFC are given in Figure 5. As expected, all three types of fiber-reinforced polymer composites showed lower densities compared to the Ref (2.35 g·cm^−3^), and as the fiber loading increased, the densities of the corresponding composites decreased, indicating that the addition of natural fibers had a positive effect on reducing the density of the composite systems [53]. Moreover, it can also be seen that, at the same fiber addition level, the density of the CDFC was slightly higher than that of the CSFC but lower than that of the SFC. When adding 3–12 wt.% of fiber loading, the density values were 2.28–2.04 g·cm^−3^, 2.25–1.99 g·cm^−3^, and 2.29–2.09 g·cm^−3^, for the CDFC, CSFC, and SFC, respectively. This may be attributed to the fact that the lower denser regions in natural fibers, such as hemicellulose and lignin, were partially decomposed under the action of cow stomach microorganisms, which resulted in a relatively increased density of the cow dung fibers as compared to the corn stalk fibers, thereby leading to a higher density of the CDFC than the CSFC [54]. In addition, the higher cellulose content in the sisal fibers contributed to the higher fiber density, which explained why the SFC exhibited the largest density among these three fiber-reinforced composites [50].

### 3.5. Water Absorption Analysis

**Figure 6:**
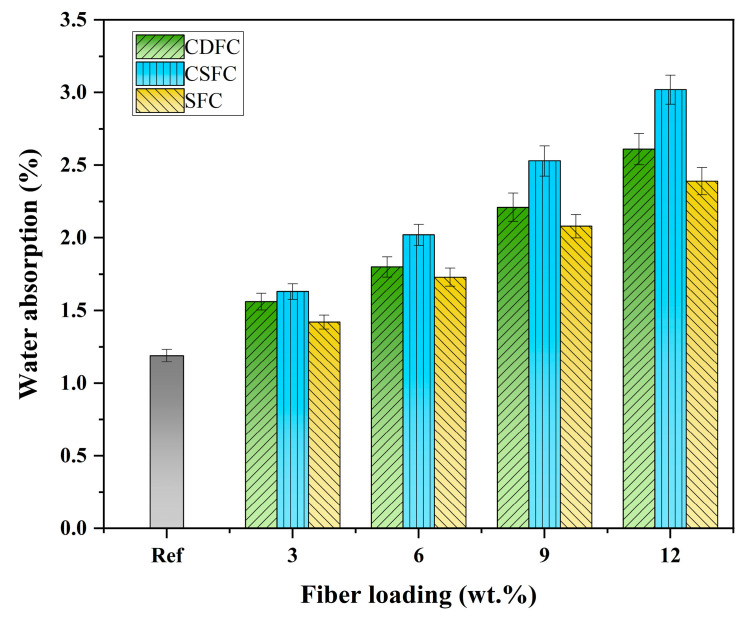
Water absorption of the polymer composites Ref, CDFC, CSFC, and SFC.

The water absorption of the polymer composites Ref, CDFC, CSFC, and SFC was measured, and the corresponding test results are given in Figure 6. The water absorption of the composites showed an increasing trend with the increase in fiber loading, where the maximum water absorption was obtained at 12 wt.% fiber addition, i.e., 2.61%, 3.02%, and 2.39% for the CDFC, CSFC, and SFC, respectively, while the minimum was found to be 1.19% for the composite Ref. This can be ascribed to the hydrophilic nature of the natural fibers; in fact, the presence of hydroxyl groups was the major contributor to the water absorption of the natural fibers, and when exposed to water, these free hydroxyl groups interacted with water molecules and formed hydrogen bonds. As the fiber loading increased, the amount of hydroxyl groups in the composite system increased, thus leading to an increase in the water uptake ability of the fiber-reinforced composites [55,56]. Moreover, according to Lou et al. [46], hemicellulose is considered to be the most hydrophilic component in natural fibers, followed by lignin, and Adhikary et al. [57] also reported that the increased crystallinity of natural fibers could reduce the water uptake of the fibrous composite, since the crystalline regions in natural fibers were impermeable to the moisture. For this study, at same fiber loading, the water adsorption of the composites with the CDF fibers was lower than those with CSF fibers but higher than those with SF fibers, and this behavior can be explained by the reasons mentioned above. This finding was consistent with the results obtained from the XRD and FTIR analyses described in Section 3.2 and Section 3.3.

### 3.6. Impact Strength Property Analysis

**Figure 7:**
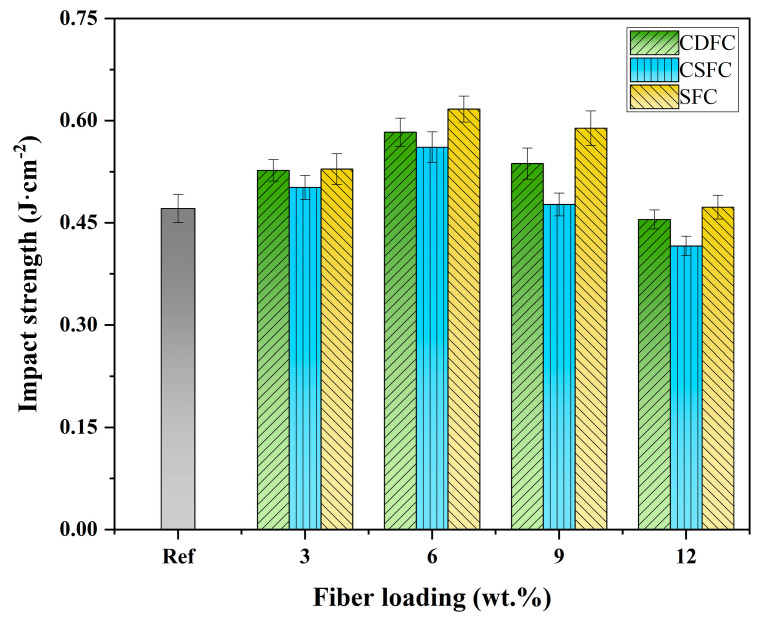
Impact strength of the polymer composites CDFC, CSFC, and SFC at different fiber loadings.

The impact strength of the polymer composites at different fiber loading levels was measured to evaluate and compare the effect of the reinforcing fibers (CDF, CSF, and SF) on the impact-resistance properties of the composite systems. The obtained impact strength results are given in Figure 7. As shown in Figure 7, the impact strength showed a similar change trend with the addition of fibers for the CDFC, CSFC, and SFC; that is, it increased with increasing fiber loading from 0 to 6 wt.%, but then decreased with a further increase in fiber addition from 6 wt.% to 12 wt.%. Among the tested composites, CDFC-6, CSFC-6, and SFC-6 exhibited the highest impact strength of 0.583 J·cm^−2^, 0.561 J·cm^−2^, and 0.616 J·cm^−2^, respectively, which were increased by 23.8%, 19.1%, and 30.8%, respectively, compared to the composite Ref (0.471 J·cm^−2^); while the lowest impact strength was observed at 12 wt.% of fiber loading, i.e., the CDFC-12, CSFC-12, and SFC-12, which were even lower than the composite without fiber addition (Ref). This can be explained by the fact that adding an appropriate content of fibers (i.e., 3 wt.% and 6 wt.% in this study) into the composite systems promoted better interfacial bonding between the fibers and composite matrix, which enabled effective stress transfer between them and limited the further propagation of the impact-induced cracks, thereby resulting in an enhancement in the impact strength. However, a higher fiber content (i.e., 9 wt.% and 12 wt.%) deteriorated fiber–matrix interfacial bonding and increased the probability of fiber agglomeration, leading to localized stress concentrations in the polymer composites, which required less energy for crack generation and propagation. Similar observations were also reported by Haque et al. [55] and Karmakar et al. [58].

Further comparing the CDFC, CSFC, and SFC, it was found that the impact strength of the CDFC was higher than that of the CSFC at all the fiber loadings, especially for the 9 wt.% loading, where it increased by 12.6%. This was mainly attributed to the rougher surface of the cow dung fibers than the corn stalk fibers (Figure 2) and the consequent enhanced interlocking action between the cow dung fibers and composite matrix. However, the CDFC had lower impact strength values as compared to the SFC, which can be ascribed to the comparatively lower crystalline cellulose content of the cow dung fibers compared with the sisal fibers (Figure 3). It was interesting to note that the impact strength of these two composites (CDFC-3 and SFC-3) was relatively close when the fiber loading was 3 wt.%. The possible reason for this behavior was that the cow dung fibers with a crinkly and even surface provided a good interfacial condition with the composite matrix, which to some extent compensated for the limitation of the crystallinity degree of the fibers themselves, with both factors significantly affecting the impact strength of the fiber-reinforced polymer composites [56,59].

### 3.7. Shear Strength Property Analysis

**Figure 8:**
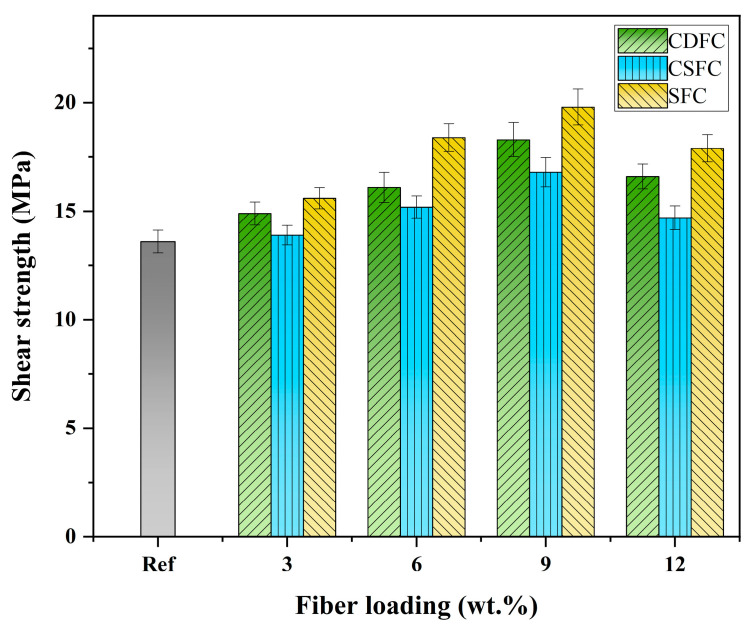
Shear strength of the polymer composites CDFC, CSFC, and SFC at different fiber loadings.

The variation in the shear strength of the polymer composites CDFC, CSFC, and SFC at different fiber loadings is illustrated in Figure 8. The inclusion of natural fibers had a positive effect on the shear strength of the composite systems, and the trend in shear strength for the CDFC, CSFC, and SFC was similar; that is, it first increased and then decreased with the increase in the fiber loading. The highest shear strength was found at 9 wt.% fiber addition, i.e., 18.3 MPa, 16.8 MPa, and 19.8 MPa for the CDFC-9, CSFC-9, and SFC-9, respectively, which were increased by 34.6%, 23.5%, and 45.6% as compared to the composite Ref. However, a further increase in the fiber loading to 12 wt.% caused a decrease in the shear strength of the composites, although it was still slightly higher than the composite without fiber addition. Such behavior was mainly due to the fibers sharing a certain shear load and demanding a higher stress for crack development, but a too high fiber loading negatively affected the fiber–matrix interfacial adhesion, thus limiting the improvement in the shear strength of the polymer composites [56,59,60].

A further comparison of the CDFC, CSFC, and SFC revealed that, at the same fiber addition level, the CDFC exhibited better shear strength compared to the CSFC but was poorer to varying degrees than the SFC. This observation was similar to the comparative results of the impact strength. In fact, as reported, the strength of the composite systems depended not only on the fiber–matrix interfacial bonding but also on the strength of the fibers [27]. The behavior of the cow dung fiber composites (CDFC) with a moderate level of shear strength can also be explained by the reasons mentioned in Section 3.6.

### 3.8. Fracture Surface Analysis

**Figure 9:**
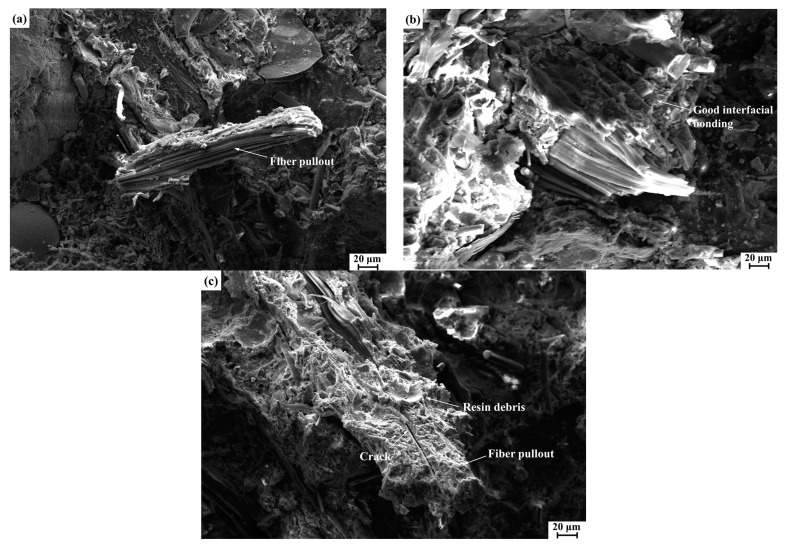
SEM images of the impact fracture surfaces of the composites at 6 wt.% fiber loading: (**a**) CSFC-6, (**b**) CDFC-6, and (**c**) SFC-6.

The fracture surfaces of the polymer composites CDFC, CSFC, and SFC after the impact and shear tests were characterized using SEM to evaluate and compare the interfacial bonding of the CDF, CSF, and SF to the composite matrix. Figure 9 shows the SEM images of the impact fracture surfaces of CDFC-6, CSFC-6, and SFC-6 (the best performance in their respective composites). For CSFC-6 (Figure 9a), the phenomenon of fiber pullout was observed on the fracture surface, and the exposed fibers had a relatively smooth surface with less resin debris attached, which indicated a weak interfacial adhesion between the corn stalk fibers and the resin matrix. For the composite CDFC-6, as shown in Figure 9b, the cow dung fibers were tightly embedded in the composite matrix and exhibited tearing and fatigue fracture instead of pullout under the applied impact load, reflecting a strong bonding quality at the fiber–matrix interface. In the case of the composite SFC-6 (Figure 9c), cracks clearly occurred in the sisal fibers, which meant that the presence of the fibers deflected the crack propagation path and dissipated more energy to resist the external impact load; moreover, fiber breakage and pullout also appeared on the fracture surface, but large amounts of resin matrix adhered to the fiber surface, revealing an acceptable fiber–matrix interfacial adhesion condition. These observations were consistent with the comparative results of the impact strength properties of these three fiber-reinforced polymer composites.

**Figure 10:**
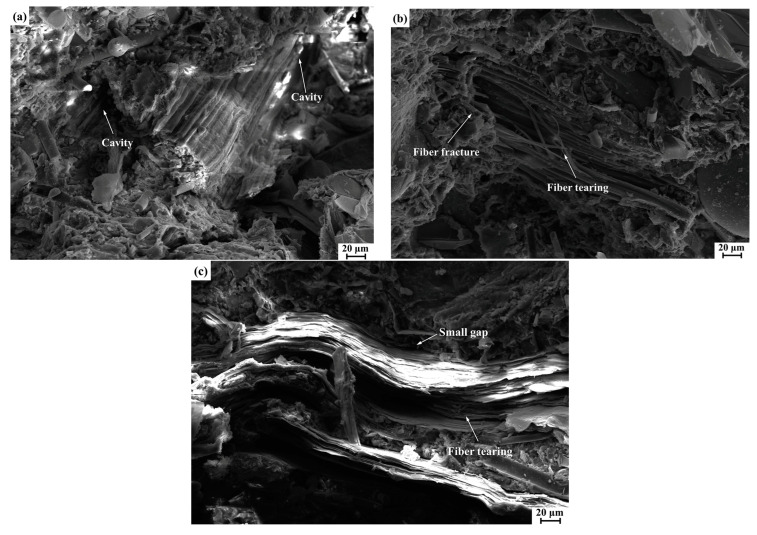
SEM images of the shear fracture surfaces of the composites at 9 wt.% fiber loading: (**a**) CSFC-9, (**b**) CDFC-9, and (**c**) SFC-9.

Figure 10 illustrates the shear fracture surfaces of CDFC-9, CSFC-9, and SFC-9 (the best performance in their respective composites). For CSFC-9, as shown in Figure 10a, the corn stalk fibers were partially debonding and pulled out under the action of the shear load, resulting in cavities left on the fracture surface. This feature suggested relatively poor interfacial adhesion of corn stalk fibers to the composite matrix. For the composite CDFC-9 (Figure 10b), a tight bonding was observed at the interface between the cow dung fibers and resin matrix, which contributed to an efficient transfer of stress between them. Moreover, fiber fracture and tearing were also observed rather than being pulled out from the composite matrix, further implying superior fiber–matrix interfacial locking. As for SFC-9 (Figure 10c), small gaps occurred at the sisal fiber–matrix interface zone, but when the composite was subjected to shear loading, the fibers were still adhered in the matrix while also exhibiting fiber tearing. It can also be clearly observed that the exposed sisal fibers had a denser structure, which corresponded to the high crystallinity of sisal fibers (Figure 3 and Table 2), and this feature also positively affected the mechanical properties of the fiber-reinforced polymer composites [27,55]. These observations were consistent with the comparative results of the shear strength properties.

## 4. Conclusions 

This work evaluated the potential use of the cow dung fibers as reinforcing material in resin-based polymer composites. The mechanical and morphological properties of the cow dung fiber composites were systematically investigated and compared with corn stalk fiber composites and sisal fiber composites. The following conclusions can be drawn:

(1) The CDF presented a rough and crinkly surface with more node structures, and their degree of crystallinity (57.69%) was higher than that of the CSF, but lower than that of the SF; 

(2) The addition of the CDF reduced the density of the polymer composites and increased the water absorption. CDFC-12 had the lowest density (2.04 g·cm^−3^) and the highest water absorption (2.61%);

(3) The incorporation of the CDF improved both the impact strength and shear strength of the polymer composites. The highest impact and shear strengths (0.583 J·cm^−2^ and 18.3 MPa) were recorded at 6 wt.% and 9 wt.% of fiber loading, respectively; 

(4) Comparative tests revealed that the CDFC exhibited better mechanical performances than the CSFC under the same fiber content; CDFC-3 showed an impact strength closer to that of SFC-3;

(5) Fracture surface analysis revealed that the CDF showed good interfacial bonding with the composite matrix and presented tearing and fatigue fracture instead of pullout under applied load. 

The above results confirm that cow dung fibers can be effectively used as reinforcing material in resin-based polymer composites, which provides an alternative application for the utilization of cow dung waste resources.

## Figures and Tables

**Table 1:** The composition of the prepared polymer composites.

Ingredients (wt.%)	Ref	CDFC				CSFC				SFC			
		3#	6#	9#	12#	3#	6#	9#	12#	3#	6#	9#	12#
Cow dung fibers	0	3	6	9	12	0	0	0	0	0	0	0	0
Corn stalk fibers	0	0	0	0	0	3	6	9	12	0	0	0	0
Sisal fibers	0	0	0	0	0	0	0	0	0	3	6	9	12
Barium sulfate	25	22	19	16	13	22	19	16	13	22	19	16	13
Parent formulation ^1^	75	75	75	75	75	75	75	75	75	75	75	75	75

^1^ The parent formulation included binder (phenolic resin 14 wt.%), modifiers (graphite 8 wt.%, antimony sulfide 3 wt.%, alumina 7 wt.%, zinc stearate 2 wt.%, porous iron powder 10 wt.%, petroleum coke 5 wt.%), fibrous ingredients (compound mineral fibers 19 wt.%), and fillers (vermiculite powder 5 wt.%, friction powder 2 wt.%).

**Table 2:** Crystallinity characteristics of the fiber samples CDF, CSF, and SF.

Fiber Samples	Peak Diffraction 2θ (°)	*CrI* (%)
CDF	15.90/22.32	57.69
CSF	16.07/22.16	51.53
SF	15.97/22.29	61.77

## Data Availability

Not applicable.
